# How β-cyclodextrin- loaded mesoporous SiO_2_ nanospheres ensure efficient adsorption of rifampicin

**DOI:** 10.3389/fchem.2022.1040435

**Published:** 2022-12-13

**Authors:** Xun Sun, Mingming Chen, Jiayu Lei, Xinran Liu, Xin Ke, Wengang Liu, Jingkuan Wang, Xiaodan Gao, Xin Liu, Yun Zhang

**Affiliations:** ^1^ Northeast Key Laboratory of Arable Land Conservation and Improvement, Ministry of Agriculture, College of Land and Environment, Shenyang Agricultural University, Shenyang, China; ^2^ Liaoning Key Laboratory of Clean Energy and College of Energy and Environmental, Shenyang Aerospace University, Shenyang, China; ^3^ School of Resources and Civil Engineering, Northeastern University, Shenyang, China

**Keywords:** β -cyclodextrin, mesoporous silica nanospheres, rifampicin, adsorption, simulated calculation

## Abstract

In this study, β-CD@mesoporous SiO_2_ nanospheres (β-CD@mSi) were prepared by loading β-cyclodextrin (β-CD) onto mesoporous silica nanospheres through an *in situ* synthesis. This not only solved the defect of β-CD being easily soluble in water, but also changed the physical structure of the mesoporous silica nanospheres. FTIR and XPS results showed that β-CD was successfully loaded onto mesoporous silica nanospheres (mSi), while enhancing the adsorption effect. β-CD@mSi with a monomer diameter of about 150 nm were prepared. At a temperature of 298k, the removal efficiency of a 100 mg/L solution of rifampicin can reach 90% in 4 h and the adsorption capacity was 275.42 mg g^−1^ at high concentration. Through the calculation and analysis of adsorption kinetics, adsorption isotherms and adsorption thermodynamics based on the experimental data, the reaction is a spontaneous endothermic reaction dominated by chemical adsorption. The electron transfer pathway, structure–activity relationship and energy between β-CD@mSi and rifampicin were investigated by quantum chemical calculations. The accuracy of the characterization test results to judge the adsorption mechanism was verified, to show the process of rifampicin removal by β-CD@mSi more clearly and convincingly. The simulation results show that π–π interaction plays a major interaction in the reaction process, followed by intermolecular hydrogen bonding and electrostatic interactions.

## 1 Introduction

Rifampicin (RIF) is a major and effective drug for the treatment of tuberculosis (Damasceno et al., 2020). The overuse and misuse of antibiotics has led to increased bacterial resistance, posing significant risks to the water resources and human health (Rodriguez-Mozaz et al., 2015). Therefore, it is necessary to find a feasible and effective technology to eliminate RIF in the water environment.

So far, many methods, such as biodegradation (Xiong et al., 2016), membrane filtration (Wang et al., 2021), photocatalytic degradation (Wang et al., 2018), AOPs (Kanakaraju et al., 2019), and adsorption (Zhao et al., 2019) have been applied to remove antibiotics from wastewater, the adsorption is considered to be an effective method among the above methods (Wei et al., 2019). Compared to other processes, adsorption has the advantages of high efficiency, low cost, regenerability and ease of operation (Mallik et al., 2022).

Traditional inorganic adsorbents are widely used in environmental remediation. Among these inorganic materials, mesoporous silica nanoparticles (MSNs) are more suitable for practical applications due to their adjustable structure (Yin et al., 2019). Grafting organics with special properties onto silicon-based materials can be considered to form new composite structure to enhance their ability to remove specific pollutants (Stephanie et al., 2012; Zhang et al., 2019). β-CD is a barrel oligosaccharides (Tian et al., 2020). Its cavity is hydrophobic inside and hydrophilic outside, due to the activity of edge hydroxyl groups, can form inclusion complexes with pollutants (Sikder et al., 2018). Kuang, silanized Fe304@SiO2 A novel magnetic surface molecularly imprinted polymer (HC/SMIPs) was prepared for the specific extraction of 4-hydroxycoumarin. The maximum adsorption capacity of HC/SMIPs could reach to 22.78 mg/g (Kuang et al., 2022). Marrane describe a novel approach for *in situ* synthesis of cellulose microfibrils-*grafted*-hydroxyapatite (CMFs-*g*-HAP_N_ (8%)) as an adsorbent using phosphate rock and date palm petiole wood as alternative and natural Moroccan resources. The maximum adsorption capacities of the CMFs-g-HAPN (8%) adsorbent toward Pb(II) and Cu(II) are 143.80 and 83.05 mg/g (Marrane et al., 2022). Since rifampicin has a similar functional group structure to the above pollutants, therefore, β-CD loaded mesoporous silica nanospheres can be used to try to remove rifampicin.

**FIGURE 1:**
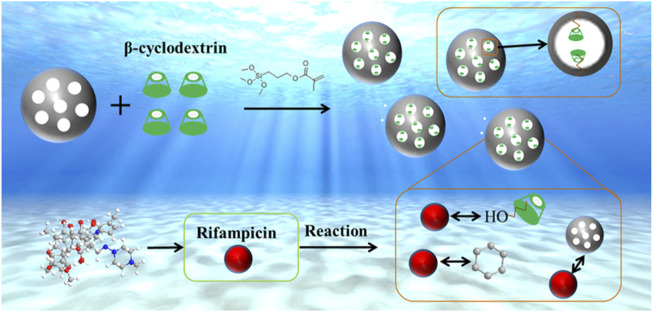
The adsorption process of RIF.

At present, there are few research on the removal of rifampicin from water environment, mainly focusing on adsorption at low concentration. The explanation of rifampicin adsorption mechanism is not sufficient. In order to solve these problems, in this study, with 3-methacryloxypropyltrimethoxysilane (KH570) as cross-linker, β-CD was loaded onto mesoporous silica nanospheres to remove RIF from aqueous solution (Figure 1). We conducted batch experiments with different factors to explore the optimal reaction conditions of RIF, and verified its adsorption capacity under high concentration. Due to the limitations of current characterization test techniques in describing electron behavior, it is difficult for these methods to provide absolutely correct results. Quantum chemical calculations can provide detailed structural and energy information (Ma et al., 2016; Liu et al., 2019), and this is necessary to study the reaction process. In order to better explain the adsorption mechanism, the density functional theory (DFT) was used as the calculation basis, the possible activation sites of β-CD and RIF were predicted by computer simulation, the electron migration path and the intermolecular interaction were verified (De et al., 2016; Khnifira et al., 2020; Mao et al., 2021). The priority of chemical reactions in the adsorption process is further elaborated, and the adsorption process of antibiotics is better explained from the electronic level.

## 2 Reagents and methods

### 2.1 Chemicals

Rifampicin was supplied by Shenyang Pharmaceutical Company. β-CD (C_42_H_70_O_35_, 98%) and cetyltrimethylammonium bromide (CTAB; C_19_H_42_BrN, 99%) were purchased from Shanghai Aladdin company. Tetraethyl orthosilicate (TEOS) was purchased from Tianjin chemical reagent factory. In this study, all chemical reagents were of analytical grade, and all solutions are prepared with pure water.

### 2.2 Composite material preparation

#### 2.2.1 Preparation of mesoporous SiO_2_ nanospheres

The whole experiment was carried out under water bath conditions (80°C). About 1 g CTAB was added to 500 ml deionized water, which was then placed in a magnetic stirrer and stirred until the solution is clear and transparent. The speed was increased and 3.5 ml of 2 mol/L NaOH solution was added, and then 5 ml of TEOS was added slowly. Stirring was continued for 2 h, and the extracted white solids were washed with deionized water for three times. After freeze-drying, it was put into a muffle furnace and calcined at 550°C in an air atmosphere for 4 h (heating rate 10°C/min) to obtain mesoporous SiO_2_ nanospheres.

#### 2.2.2 Preparation of β-CD@mSi

Mesoporous silica powder suspension (pH = 4) was prepared and placed in a magnetic stirrer for stirring, while adding 1 ml KH570 and 20 ml β-CD solution (pH = 4). It was stirred and allowed to react for 6 h to obtain β-CD@SiO_2_ nanospheres.

### 2.3 Characterizations

The specific surface area and pore diameter of mSi and β-CD@mSi were measured using a V-sorb 2,800 analytical tester. The morphology and structure of mSi and β-CD@mSi were observed by a S-4800 (Hitachi, Japan) SEM. The element distribution of β-CD@mSi was investigated using a Tecnai G2 F20 TEM (FEI, Hillsboro, OR, United States). Changes in elemental composition before and after β-CD@mSi adsorption were investigated using a ESCALAB 250 XI analyzer (Thermo, Waltham, MA, United States). IR was analyzed using a Nicolet 460 spectrometer (Thermo Fisher, Waltham, MA, United States).

### 2.4 Adsorption experiments

The effects of β-CD@mSi on RIF adsorption were studied by batch experiments under different conditions, including the amount of β-CD@mSi (10–50 mg), time (1–6 h), initial concentration of RIF (100–700 mg L^−1^), pH (2–7), and temperature (288–308 K). Adjust the pH of the solution with 0.1 mol L^−1^ NaOH and HCl solution. A fixed amount of β-CD@mSi was added in 50.0 ml RIF solution. A series of adsorption experiments was carried out on a thermostatic oscillator (rotation frequency: 180 rpm). Residual RIF concentration was measured at the maximum absorbance (λ _Max_) of 474 nm using an UV spectrophotometer (Hosseini et al., 2020). In order to guarantee the accuracy of experimental results, all test samples were tested in triplicate.

The adsorption capacity (qt) per Gram of β-CD@mSi and the removal efficiency of RIF at time t are calculated as follows: 1) The adsorption capacity of RIF; 2) Removal efficiency of RIF.

$$q_{t}=\frac{\left(C_{0}-C_{t}\right)V}{M}$$ (1)

$$RIF\;removal\left(\%\right)=\frac{\left(C_{0}-C_{t}\right)}{C_{0}}\times 100$$ (2)

where C_0_ is initial concentration (mg·L^−1^); C_t_ is the concentration at completion of adsorption (mg·L^−1^); V is the volume of RIF solution (L); M is the mass of adsorbent (g).

### 2.5 Quantum chemical calculation

Simulation calculations were performed using the Dmol3 module in Material Studio 2019. After structure optimization, then DNP 3.5 basis set was selected for calculation, and PBE of generalized gradient approximation (GGA) was selected for functional (Ghahghaey et al., 2020; Cao et al., 2021). In energy optimization, the SCF tolerance is 1.0 × 10^–6^, the smearing is 0.005 Ha, water was used as solvent, and the dielectric constant is 78.54. According to density functional theory, HOMO, LUMO, and electrostatic potential were calculated (Tanzifi et al., 2020; Chen et al., 2021). The adsorption binding energy E_ads_ was calculated according to Eq. 3.

$$E_{ads\;}=E_{total}-E_{absorbate}-E_{adsorbent}$$ (3)

where E_total_ is the Total energy, E_adsorbate_ is the energy of rifampicin after optimization and E_adsorbent_ is the energy of β-CD@mSi after optimization。

## 3 Results and analysis

### 3.1 Characterization of materials

#### 3.1.1 SEM and BET analysis

**FIGURE 2:**
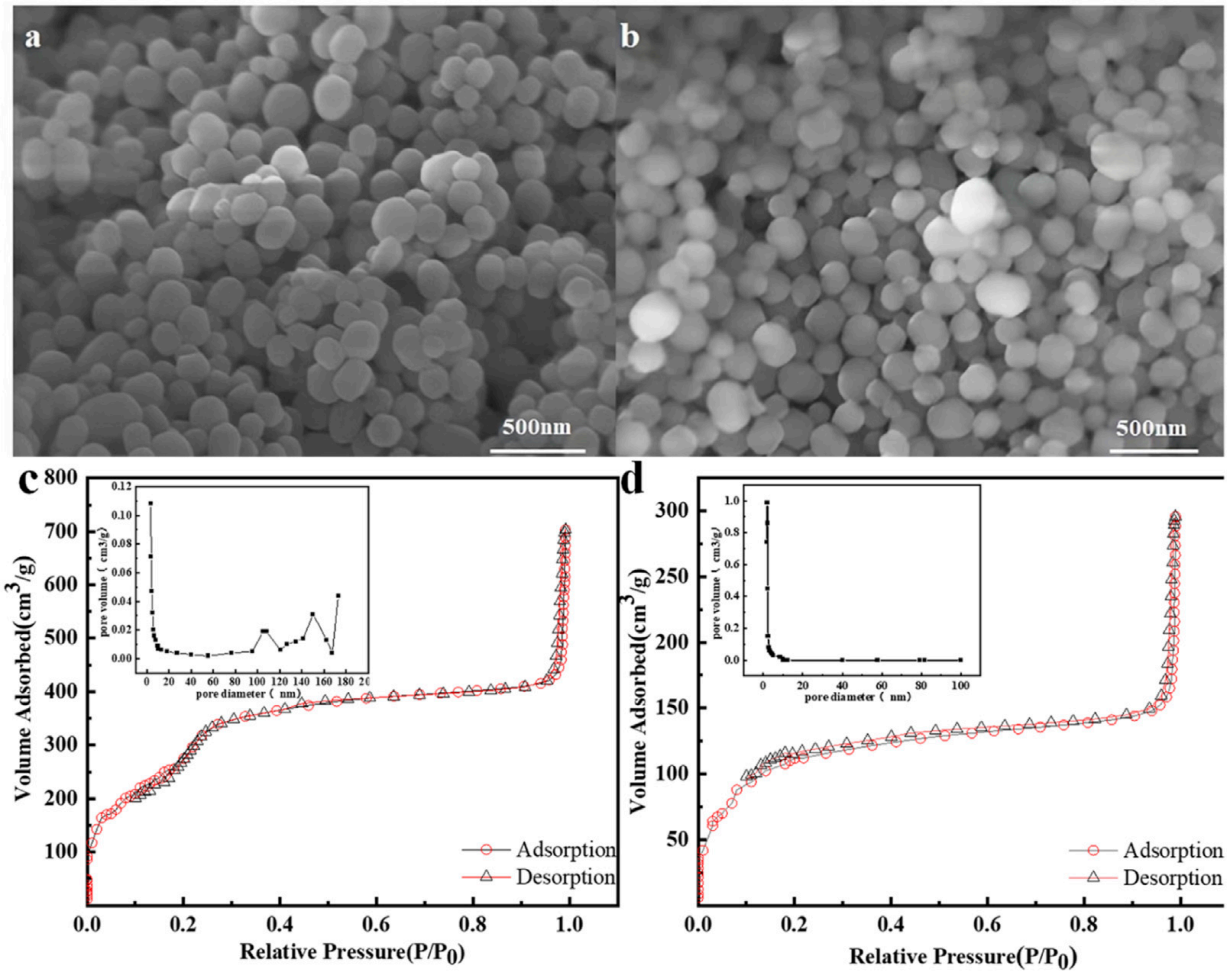
**(A)** SEM image of mSi; **(B)** SEM image of β-CD@mSi; **(C)** Nitrogen adsorption-desorption isotherm for mSi; **(D)** Nitrogen adsorption-desorption isotherm for β-CD@mSi.

The exterior morphology of mSi and β-CD@mSi were characterized by SEM, as shown in Figure 2A. The mSi has a smooth spherical shape and the diameter ranges from 100 to 150 nm. After loading with β-CD, the size of the composite material did not change significantly (Figure 2B). The N_2_ adsorption and desorption isotherms of mSi and β-CD@mSi correspond to the type IV isotherm. The N_2_ adsorption capacity of mSi and β-CD@mSi increases rapidly in the low pressure region, and remains relatively fixed in a wide pressure range with the increasing relative pressure, indicating that there are micropores and mesoporous pores in both mSi and β-CD@mSi. The BET surface area of mSi is 1,151.41 m^2^/g, the total pore volume is 0.97 cm^3^/g, and the average aperture size is 4.79 nm. After the β-CD is coated, the specific surface area of β-CD@mSi (418 m^2^/g) and total pore volume (0.32 cm^3^/g) decreased significantly. This indicates that β-CD enters the internal pore channels of the mesoporous silica nanospheres, thereby reducing the specific surface area and total pore volume (Neeli et al., 2018).

#### 3.1.2 TEM analysis

In Supplementary Figure S3, TEM images of β-CD@mSi show that it is mainly composed of C, O, Si, and the distribution of C, O, and Si is uniform, indicating that β-CD@mSi is well prepared. The diameter of the monomeric particles of β-CD@mSi is around 150 nm, which is consistent with the SEM results. In addition, we also found that some C and O elements were distributed outside the blue spherical region of C and the green spherical region of O, indicating that β-CD was successfully loaded onto the surface of mSi.

#### 3.1.3 XPS analysis

The element composition of β-CD@mSi surface before and after adsorption can be analyzed using XPS. The XPS spectra before and after adsorption of β-CD@mSi are shown in Supplementary Figure S4. The material has three elements, C, O and Si, which is consistent with the elemental composition of the material itself. Before adsorption, three peaks were fitted to the C1s spectrum at 284.8, 286.39 and 288.75 eV, corresponding to the C-C, C-O and O-C=O groups respectively (Zheng et al., 2018). Peaks fitted to the O1s spectrum at 532.41 eV and 533.27 eV correspond to C-O and C=O, which indicates that the composite The presence of oxygen-containing functional groups. The area corresponding to the C-C group and O=C-O group decreased and the area of the C-O group increased after adsorption. The peaks fitted to the O1s spectra at 532.41 eV and 533.27 eV shifted to 532.55 eV and 533.57 eV, corresponding to a decrease in the area of the C-O group and an increase in the area of the C=O group, which is related to the adsorption of rifampicin on the β-CD@mSi (Zhang et al., 2016). Following RIF adsorption, the N1s spectrum (Supplementary Figure S4D), showed a new fitted peak at 399.68 eV, which corresponded to the C-NH2 group on rifampicin, indicating that RIF had successfully adsorbed onto β-CD@mSi.

#### 3.1.4 FTIR analysis

As shown in Supplementary Figure S5, the characteristic peaks of β-CD@mSi at 1,092, 1,635, 1700, and 3,440 cm^−1^ correspond to C-O, C-O-C, C-C, and O-H bonds of the composite materials, respectively. The appearance of the characteristic peaks of β-CD (C-O-C) indicates the successful loading of β-CD. The peak at 1,635 cm^−1^ represents the stretching vibration of the aromatic ring of β-CD. The infrared spectrum after adsorption has an obvious characteristic peak at 1,699 cm^−1^ and the intensity of the peak is obviously weakened, which corresponds to the stretching vibration of the amide bond on rifampicin, indicating that rifampicin was successfully adsorbed to β-CD@mSi (Yin et al., 2019).

### 3.2 Experimental influence parameter

#### 3.2.1 Effect of adsorbent dosage

The removal rate of rifampicin increased with the increase of the dosage of β-CD@mSi, and the removal rate reached 90.95% at 0.8 g L^−1^. As displayed in Supplementary Figure S6A, the results showed that with the increase of β-CD@mSi dose, the number of adsorption sites increased. The constant concentration of RIF solution leads to the decrease of adsorption capacity. Under the premise of ensuring the removal efficiency, 0.8 g L^−1^ was selected as the dosage.

#### 3.2.2 Effect of pH

The removal efficiency of rifampicin was greatly affected by pH (Supplementary Figure S6B). The adsorption capacity of RIF decreased, when the pH gradually increased. According to zeta potential, when pH is between 1 and 6.1, rifampicin surface is still dominated by positive charge. When pH is between 6.1 and 7, the surface is dominated by negative charge. Since RIF has two pkas (1.7 and 7.9), when pH = 2, the ionization degree of RIF is the best, and removal of RIF is mainly through π–π interactions and hydrogen bonding. β-CD@mSi and RIF are electropositive, so the electrostatic attraction ability is very weak. It has been proved that intermolecular hydrogen bond strength decreases with the increase of pH (Rodriguez-Mozaz et al., 2015). With the increase of pH, the intermolecular hydrogen bonding is weakened, and the adsorption capacity decreases gradually. When pH = 7, both β-CD@mSi and RIF are electronegative, so the electrostatic attraction weakened and the adsorption capacity further decreased.

### 3.3 Adsorption kinetics

Pseudo-first-order model (Supplementary Equation S4), pseudo-second-order model (Supplementary Equation S5), Elovich model (Supplementary Equation S6), and Weber–Morris particle intra diffusion model (Supplementary Equation S7) were used to analyze the adsorption kinetics. The detailed calculation formula can be found in the supplementary literature.

The fitting results of β-CD@mSi are shown in Supplementary Figure S7. It can be found in Table 1 that the R^2^ value of the quasi-second-order kinetic model is more in line with the experimental situation. Elovich model fitting results show that the adsorption process of RIF is chemisorption (Pandey et al., 2015). According to the intra-particle diffusion model, due to the large concentration difference between β-CD@mSi and RIF, we found the first line is steeper and can be thought to be the migration of RIF to the outer surface of β-CD@mSi by membrane diffusion. The second line is the intraparticle diffusion process. The third line shows adsorption equilibrium and its slope is relatively small. It is clear that the straight line in the first stage does not pass through the origin, membrane diffusion occurs simultaneously with intraparticle diffusion throughout the adsorption process (Wu et al., 2014; Asgharzadeha et al., 2019).

**TABLE 1:** Fitting results of adsorption kinetic model.

Kinetic models	Parameters	Temperature		
		288K	298K	308K
Pseudo first-order	q_e_	99.69	106.81	123.37
	K_1_	2.14	1.65	1.82
	R^2^	0.99	0.991	0.998
Pseudo second-order	q_e_	105.02	115.35	130.95
	K_2_	0.05	0.02	0.03
	R^2^	0.995	0.998	0.999
Elovich	R^2^	0.952	0.955	0.994

### 3.4 Adsorption isotherms

Description of adsorption isotherms by using the Langmuir model (Supplementary Equation S8), the Freundlich model (Supplementary Equation S9), and the Temkin model (Supplementary Equation S10). The detailed calculation formula can be found in the supplementary literature.

Supplementary Figure S8 is the fitting result of the adsorption isotherm model of β-CD@mSi. The theoretical adsorption capacity calculated by Langmuir is slightly different from the experimental result of RIF, and the fitting degree is poor. From the comparison of R^2^ values (Table 2), it can be clearly seen that the Freundlich model is more in line with the process description, indicating that the adsorption of rifampicin by β-CD@mSi is a multilayer adsorption. Temkin isotherms describe the chemisorption of the adsorbent on the adsorbed material, confirming that the adsorption process is chemisorption.

**TABLE 2:** Fitting results of the isotherm model of β-CD@mSi.

Isothermal models	Parameters	Temperature (K)		
		288	298	308
Langmuir	*q*_max_	254.78	273.32	388.15
	*K*_L_	0.003	0.026	0.043
	R^2^	0.416	0.709	0.78
	n	3.378	4.292	5.181
Freundlich				
	*K*_F_	47.44	73.89	130.61
	R^2^	0.913	0.98	0.993
Temkin	*K*_T_	34.66	39.91	49.21
	*f*	7.89	1.45	0.05
	R^2^	0.869	0.966	0.990

### 3.5 Adsorption thermodynamics

The detailed calculation formula of adsorption thermodynamics can be found in the supplementary literature. The Freundlich constant (K_F_) is used to derive the equilibrium constant (Kc) (Tran et al., 2017), and the calculation results are shown in Table 3. As the temperature increases, the results for ΔG_0_ are in the order -9.24, -10.66 and -12.48 kJ mol^−1^. This indicates that RIF is more favorable at β-CD@mSi at high temperature, as the temperature rises, the adsorption effect of β-CD@mSi gets better. ΔH_0_ = 43.47 kJ mol^−1^. It indicates that the adsorption process is chemical adsorption (Tran et al., 2016). The adsorption of RIF at β-CD@mSi is a spontaneous endothermic process.

**TABLE 3:** Thermodynamic parameters.

Contaminant	ΔH_0_ (kJ.mol^−1^)	ΔS_0_ (kJ.mol^−1^)	ΔG_0_ (kJ.mol^−1^)		
			288K	298K	308K
rifampicin	43.47	0.22	−9.24	−10.66	−12.48

### 3.6 Removal mechanism of rifampicin by β-CD@mSi

**FIGURE 3:**
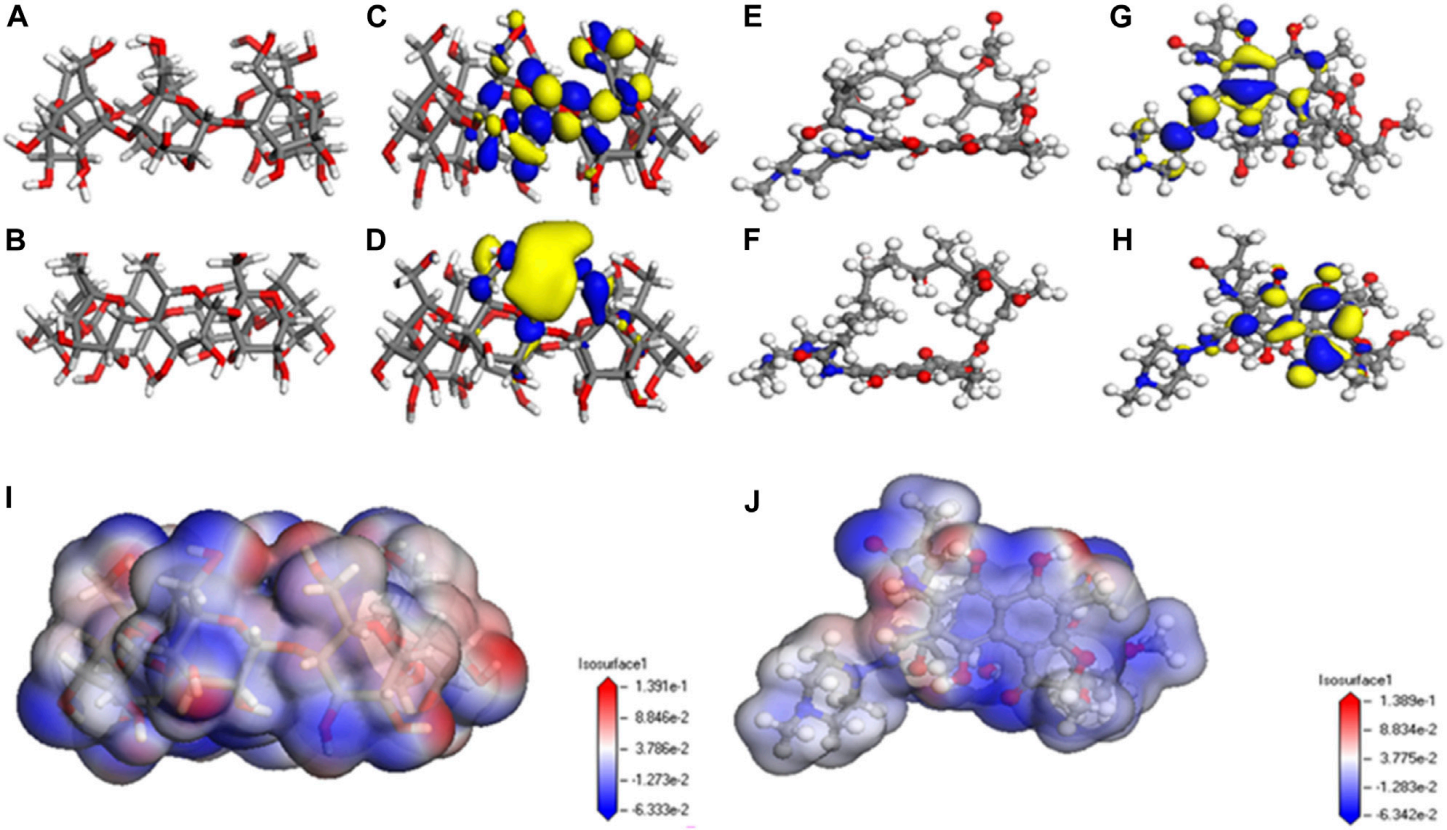
Simulation results of β-CD@mSi and rifampicin; **(A)** structure before optimization of β-CD@mSi; **(B)** the optimized structure of β-CD@mSi; **(C)** the highest occupied molecular orbital of β-CD; **(D)** the lowest unoccupied molecular orbital of RIF; **(E)** structure before optimization of RIF; **(F)** the optimized structure of RIF; **(G)** the highest occupied molecular orbital of RIF; **(H)** the lowest unoccupied molecular orbital of RIF; **(I)** surface electrostatic potential of β-CD; **(J)** surface electrostatic potential of RIF.

**FIGURE 4:**
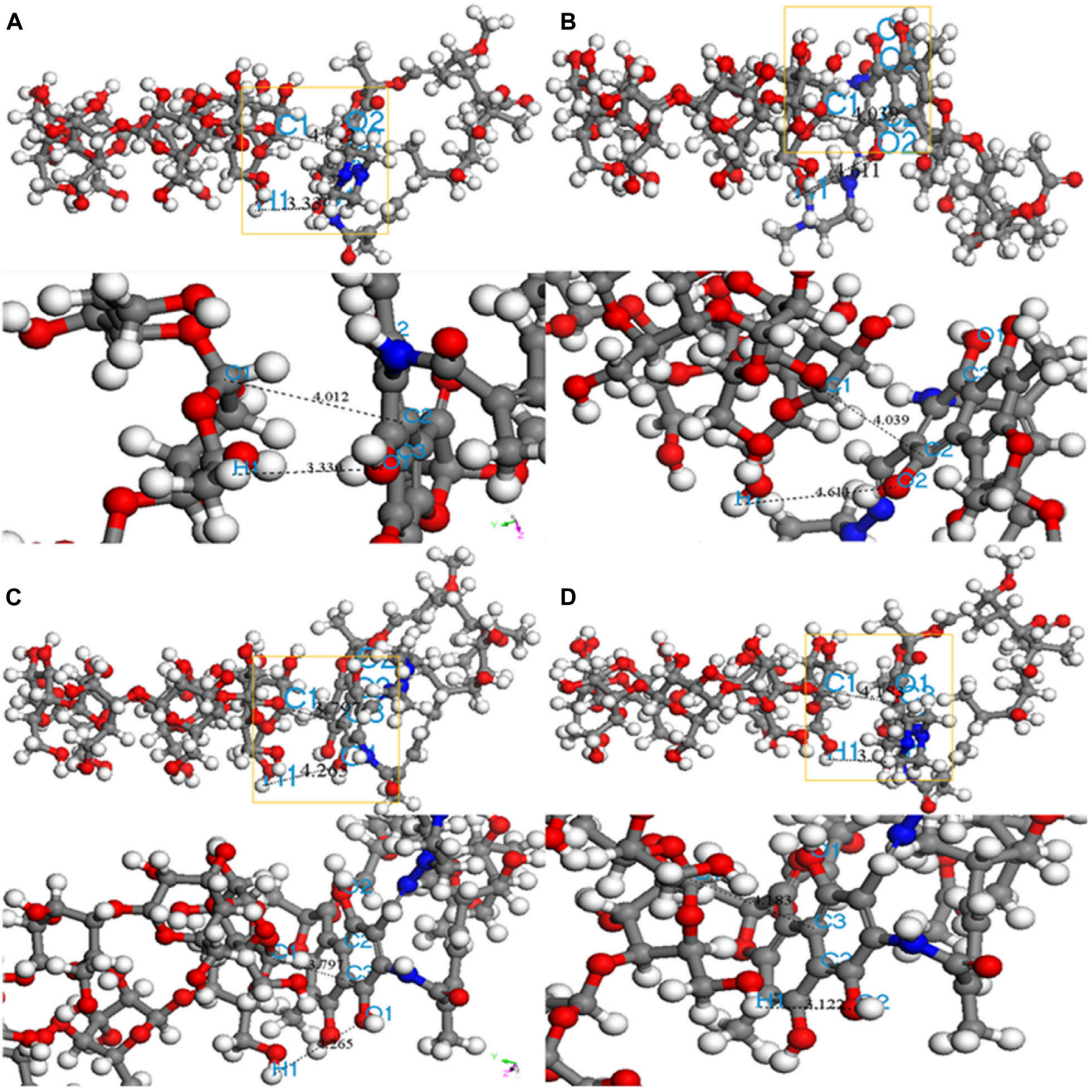
Four configurations of RIF aided on β ‐CD@mSi **(A)** C1‐C2 H1‐O1 adsorption configuration; **(B)** C1‐C2 H1‐O2 adsorption configuration; **(C)** C1-C3 H1‐O1 adsorption configuration; **(D)** C1‐C3 H1‐O2 adsorption configuration.

Adsorption kinetics and isotherm results show that the adsorption of β-CD@mSi to RIF may be mainly chemisorption. According to the analysis of infrared spectroscopy and the results of XPS, we believe that the main adsorption mechanisms are π–π interaction between aromatic rings, intermolecular hydrogen bonding, and intermolecular electrostatic interaction. In order to explain the adsorption mechanism in more depth, we use the computer to simulate and calculate, and first optimize the structure of β-cyclodextrin and rifampicin (Safia et al., 2019; Liu et al., 2004). As shown in Figures 3B,F the cavity of the optimized rifampicin ring structure is obviously enlarged and the structure is more stable. According to density functional theory and frontier orbital theory, the HOMO and LUMO orbitals of β-cyclodextrin and rifampicin are calculated respectively, as shown in Figures 3C,D,G,H, the electron loss region of β-CD is mainly concentrated in the six-membered ring, and the electron gain region is concentrated in the hydroxymethyl group. The area where rifampicin gains and loses electrons is concentrated in the benzene ring and its adjacent aromatic ring. In contrast, the hydroxyl group on the ring is easier to get electrons. The electronegativity (χ) obtained from orbital calculations shows that the χ value of β-CD is −0.10041 Ha, and the χ value of RIF is −0.12475 Ha. Since electrons will flow from lower electronegativity to higher electronegativity, it indicates that the transfer direction of electrons is from β-CD to RIF. According to the previous judgment on the adsorption mechanism and the electron gain and loss area, we believe that the six-membered ring on β-CD interacts with the benzene ring on rifampicin. The hydrogen bonds are formed between the hydroxyl group on rifampicin and the C-H bond on β-CD. As shown in Figure 4, according to the surface electrostatic potential of β-CD and rifampicin, the surface potential of C atom near O atom on β-CD is positive, and we name this point C1. The surface potential of the benzene ring and the adjacent aromatic ring of rifampicin is negative, and the C atoms at the junction of the two rings are named C2 and C3, respectively. The surface potential of the hydroxymethyl group of β-CD is positive, and the H atom on the hydroxyl group is named H1. The surface potential of the hydroxyl groups on both sides of the benzene ring on rifampicin is negative, and the O atoms on the hydroxyl groups are named O1 and O2, respectively. We carried out the corresponding structure construction and simulation calculation, essentially adjusting the distance between hydrogen bonds and π–π. The calculation results are shown in Table 4. After calculation, structure C has the lowest binding energy, indicating that its overall structure is the most stable. We consider this structure to be the most stable configuration for rifampicin adsorption on β-CD@mSi (Chen et al., 2019; Yao et al., 2019; Chen et al., 2020). The distance between aromatic rings is 3.797 Å, and the π–π interaction was predominant in the reaction process. The intermolecular hydrogen bond distance of this configuration exceeds 3 Å, indicating the existence of weak hydrogen bonds between molecules. We therefore believe that π–π interaction was predominant in the reaction process, followed by intermolecular hydrogen bonding and electrostatic interactions (Hosseini et al., 2020).

**TABLE 4:** Binding energy results for the four corresponding structures.

Structure	Corresponding locus	Total energy (kcal/mol)	Binding energy (kcal/mol)
A	C1-C2;H1-O1	419.256	−17.660
B	C1-C2;H1-O2	418.667	−18.249
C	C1-C3;H1-O1	406.243	−30.673
D	C1-C3;H1-O2	422.769	−14.147

### 3.7 Other adsorbents

As shown in Table 5, we compared β-CD@mSi with other rifampicin adsorption materials in the past 5 years, its adsorption capacity has been greatly improved. In addition to the high specific surface area of β-CD@mSi, the abundant hydroxyl functional groups of β-CD provide more reactive sites for the adsorption of rifampicin, which enables β-CD@mSi to remove rifampicin not only through electrostatic attraction. This study shows that β-CD@mSi is a promising environmental material for antibiotic removal.

**TABLE 5:** Comparison of different adsorbents.

Adsorbent	Pollutants	Adsorption capacity (mg.g^−1^)	Reference
Fe-NPs	Rifampicin	107.7	Lin et al. (2020)
Fe_3_O_4_	rifampicin	84.8	Cai et al. (2019)
rGO@Fe/Pd-NPs	rifampicin	90.9	Xu et al. (2020)
GO/CS/Fe_3_O_4_	rifampicin	101.22	Shafaati et al. (2020)
β-CD@mSi	rifampicin	246.75	This work

## 4 Conclusion

In this study, the *in situ* synthesis method was used to prepare β-CD@mSi, and the removal rate of rifampicin reached 90% in 298 K. The thermodynamic results show that the adsorption of rifampicin is a spontaneous endothermic chemical process. The results of adsorption isotherm show that RIF is multilayer adsorption on β-CD@mSi. Through computer simulation, The addition of β-CD enhances the π-π interaction between β-CD@mSi and RIF, thus enhancing the removal effect.π–π interaction is the dominant role in the adsorption process, followed by intermolecular hydrogen bonding and electrostatic attraction. Our research will contribute to the study of the preparation of environmental materials for RIF removal, β-CD@mSi has excellent environmental remediation properties and is suitable for future applications in wastewater treatment Bhattarai et al., 2014, Howes et al., 2007, Yin et al., 2019.

## Data Availability

The original contributions presented in the study are included in the article/Supplementary Material, further inquiries can be directed to the corresponding author.
