# How β-cyclodextrin- loaded mesoporous SiO_2_ nanospheres ensure efficient adsorption of rifampicin

**DOI:** 10.3389/fchem.2023.1157007

**Published:** 2023-03-10

**Authors:** Xun Sun, Mingming Chen, Jiayu Lei, Xinran Liu, Xin Ke, Wengang Liu, Jingkuan Wang, Xiaodan Gao, Xin Liu, Yun Zhang

**Affiliations:** ^1^ Northeast Key Laboratory of Arable Land Conservation and Improvement, Ministry of Agriculture, College of Land and Environment, Shenyang Agricultural University, Shenyang, China; ^2^ Liaoning Key Laboratory of Clean Energy and College of Energy and Environmental, Shenyang Aerospace University, Shenyang, China; ^3^ School of Resources and Civil Engineering, Northeastern University, Shenyang, China

**Keywords:** β –cyclodextrin, mesoporous silica nanospheres, rifampicin, adsorption, simulated calculation

In the original article, there were errors in the affiliation numbering and the affiliation numbers assigned to each author. The correct affiliations appears above.

The authors apologize for these errors and state that this does not change the scientific conclusions of the article in any way. The original article has been updated.

