# Supplementary material for: How β-cyclodextrin- loaded mesoporous SiO2 nanospheres ensure efficient adsorption of rifampicin
Source: Front Chem. 2022 Dec 13;10:1040435. doi: 10.3389/fchem.2022.1040435 (PMC9794459; doi:10.3389/fchem.2022.1040435)
Supplement: Supplementary file 1 [file DataSheet1.docx]

**Data supplement**

**3.** **TEM analysis**

**
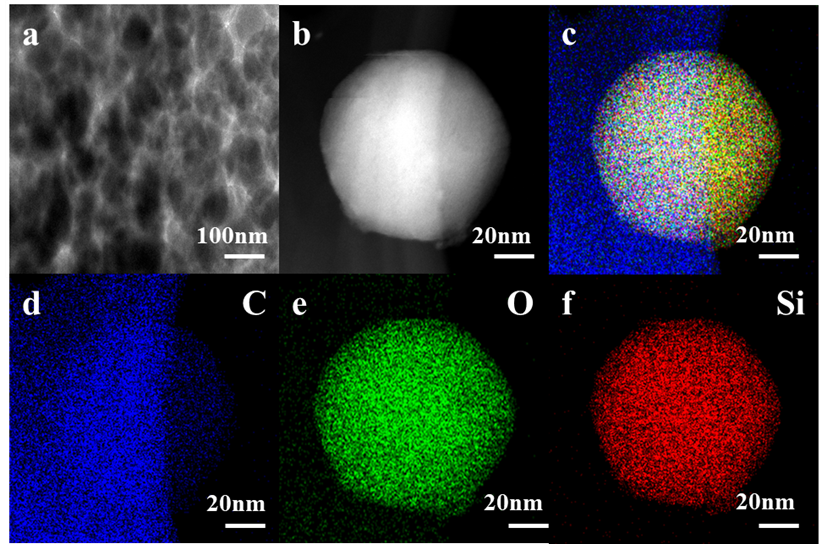
**

Fig.3. TEM image of β-CD@mSi.

**4. XPS analysis**

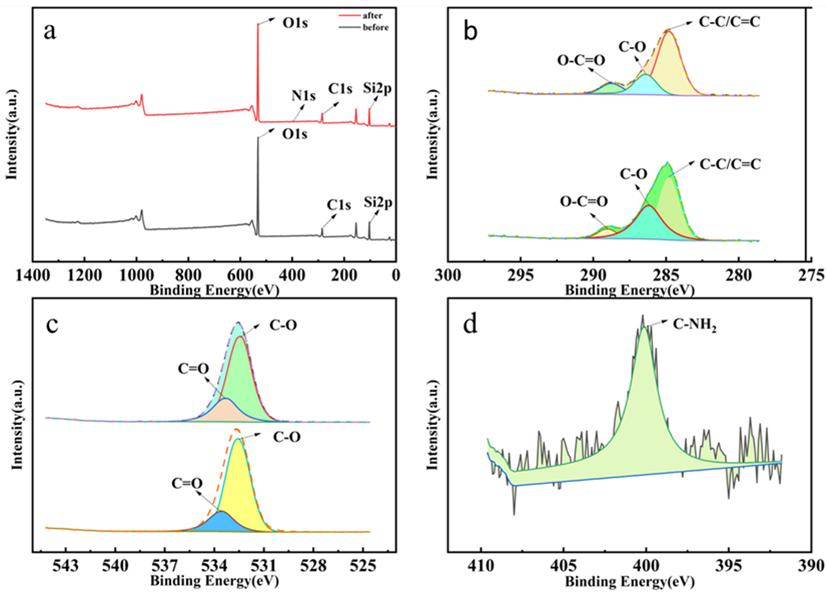


Fig.4. Full XPS spectra of β-CD@mSi before and after adsorption (a); C1s core level spectra of β-CD@mSi before and after adsorption (b); O1s core level spectra of β-CD@mSi before and after adsorption (c); N1s core level spectra of β-CD@mSi after adsorption (d).

**5. FTIR analysis**

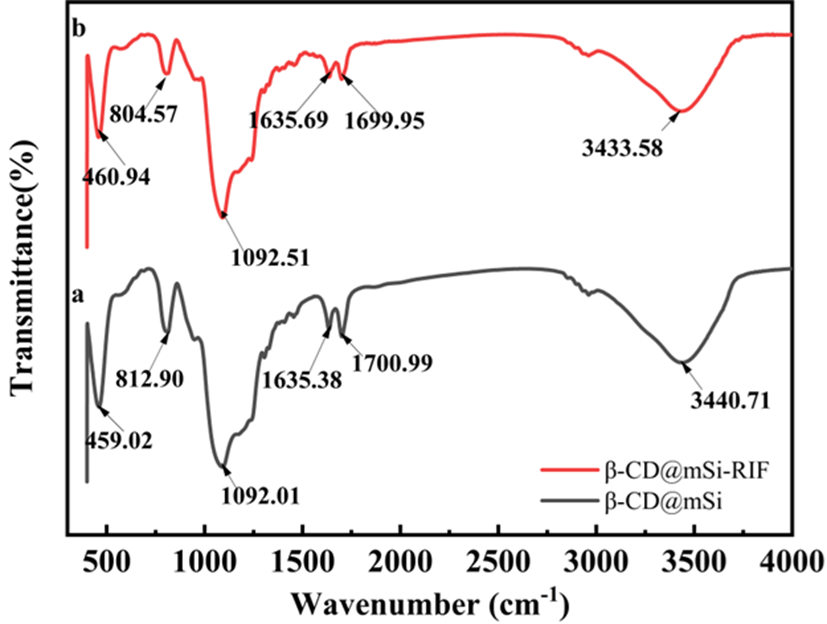


Fig.5. (a) FTIR image of β-CD@mSi; (b) FTIR image of β-CD@mSi after adsorption.

6.

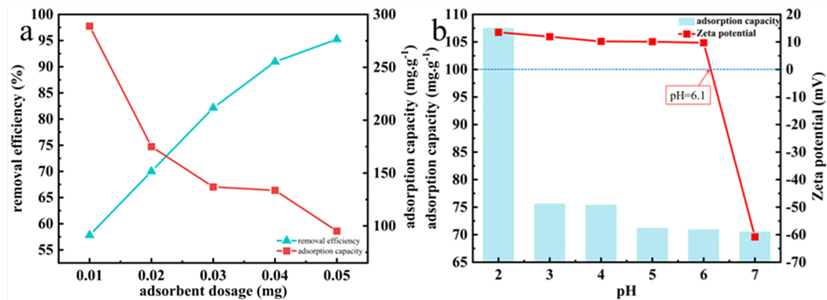


Fig.6. ( a) The effect of adsorbent dosage; (b) the effect of pH.

**7. Adsorption kinetics**


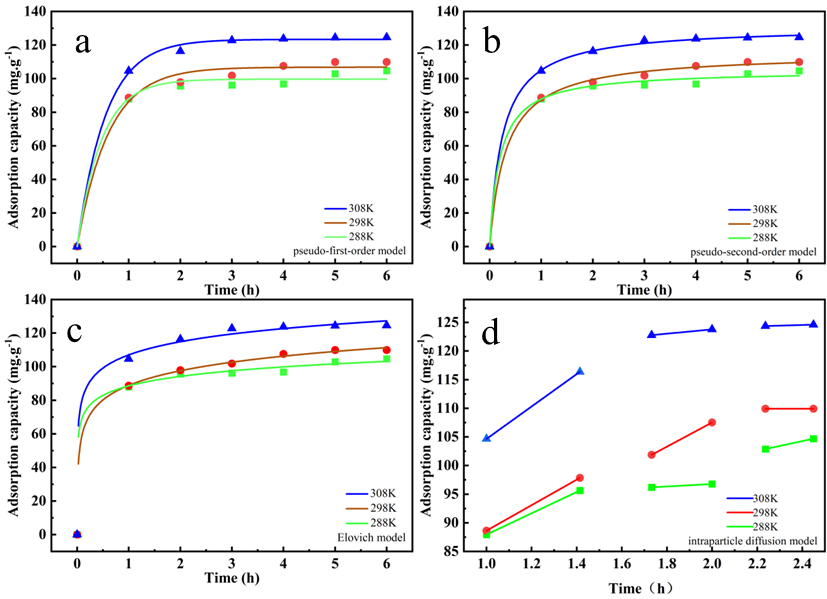


Fig.7. Adsorption kinetics experiment of β-CD@mSi and adsorption kinetics fitting.(a) pseudo-first-order adsorption kinetic model; (b) pseudo-second-order adsorption kinetic model; (c) Elovich model; (d) Weber-Morris intraparticle diffusion model.

Pseudo-first-order model (Eq.4), pseudo-second-order model (Eq.5), Elovich model (Eq.6) and Weber-Morris particle intra diffusion model (Eq.7)

$$q_{t}=q_{e}\left( 1-e^{{-k}_{1}t} \right) (4)$$

$$q_{t}=\frac{k_{2}q_{e}^{2}t}{1+k_{2}q_{e}t} (5)$$

$q_{t}=\frac{1}{\beta}\ln\left( \alpha\beta\right)+\frac{1}{\beta}\ln\left( t \right) (6)$

$q_{t}=K_{i}t^{1/2}+C (7)$

where q_t_ (mg.g^-1^)is the amount of adsorbed RIF at time t (h); q_e_ (mg.g^-1^) is the amount of adsorbed RIF at equilibrium; K_1_ and K_2_ are the rate constant of pseudo-first-order kinetic model and pseudo-second-order kinetic model respectively; α is initial adsorption rate constant, β is desorption rate constant；K_i_ is the intraparticle diffusion rate constant; and C is the intercept related to the thickness of the boundary layer.

**8. Adsorption isotherms**

**
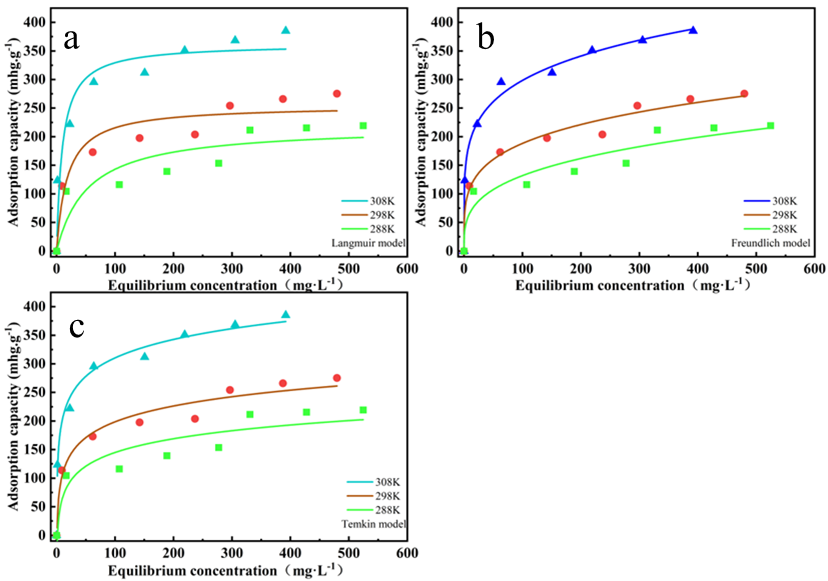
**

Fig.8. Adsorption isotherm experiment of β-CD@mSi and the fitting result of adsorption isotherm model. (a) Langmuir model (b) Freundlich model (c) Temkin model

Langmuir model (Eq. 8), the Freundlich model (Eq. 9) and the Temkin model (Eq. 10) .

$$q_{e}=\frac{q_{m}K_{L}C_{e}}{1+K_{L}C_{e}} (8)$$

$$q_{e}=K_{F}{C_{e}}^{1/n} (9)$$

$q_{e}=K_{T}\ln\left( fC_{e} \right) (10)$

where q_e_ (mg·g^-1^) is the amount of contaminants adsorbed at the time of equilibrium, q_m_ is the theoretical maximum adsorption capacity (mg·g^-1^), C_e_ (mg·L^-1^) is the concentration of residual contaminants in solution at equilibrium, and K_L_ (L·mg^-1^) is the Langmuir constant. K_F_ (mg·mg^-1^) is the binding energy constant, *K*_T_ (J/mol) and *f* (L/ mg) are the Temkin constant and the Temkin binding constant.

**9. Adsorption thermodynamics**

$$\ln\frac{q_{e}}{C_{e}}=\frac{{\Delta S}_{0}}{R}-\frac{\Delta H_{0}}{RT} (11)$$

$$K=\frac{q_{e}}{C_{e}} (12)$$

${\Delta G}_{0}=-RTlnK$(13)

Where, ΔS_0_ is entropy change (kJ.mol^-1^ ); ΔH_0_ is enthalpy change (kJ.mol^-1^ ); ΔG_0_ is Gibbs free energy (kJ.mol^-1^); R is the universal gas constant (8.314 J.mol.K^-1^).
